# Cheoplasty

**Published:** 1885-01

**Authors:** Lawrence Vanderpant


ARTICLE IX.

### CHEOPLASTY.

BY LAWRENCE VANDERPANT, L. D. S., ETC.

This term, derived from two Greek words, signifying literally, "*to fashion by pouring*," was, some twenty-five years since, applied by Dr. Blandy of Baltimore, to a system of constructing artificial dentures by means of a casting of an alloy of tin by a process very similar to the now well-known rubber process, for which he obtained two or more patents. For several reasons the system did not receive popular favor, perhaps the chief one being the advent of rubber about the same period, and, may be, the inventor claiming too much, and urging a universal adoption of his method to the exclusion of gold and other material. In various forms, at different times, it has been brought to professional notice under the name of "*Adamantine*," *Weston's Metal*, and more recently under the auspices of Drs. Atkinson and Reese by that of "*Cast Gold Alloy*," and undoubtedly these gentlemen have, by the bestowal of care, patience, and scientific application, produced a result far in advance of anything hitherto achieved, and the advocacy of the former, for its therapeutic merits, has rendered it a *sine qua non* to every liberal and advanced practitioner in dental prosthesis.

Although the manipulative process is by far the most simple in Dental Mechanics, yet it is one in which failure and disgust will most frequently occur in early experiment. The writer gave earnest attention to the subject some twenty-three years since, but from repeated failure abandoned it, and it was not until he saw the splended results

(to the patient) in the offices of the above-named gentlemen, that he again experimented, with the view of affording the profession a positive, unerring means of producing a cast metal base, resulting in an apparatus designated *The Positive Cheoplastic Fabricator,** previous to which *The Campbell New Mode Heater* had afforded a most efficient auxiliary, (by making some simple additions,) but it was evident a less costly and more simple apparatus was needed.

Excepting Dr. Reese's method, the only means at hand are different descriptions of flasks, one of which was patented by Dr. Lawrence, of Lowell, Mass, others by Drs. Weston, Moffat and Watts, but as failure is more likely than not to occur by their use, they are unpopular—"*it is as essential to regulate the Heat in Cheoplasty as in Vulcanizing.*" Such premise being granted, it will be readily understood that the ordinary Cheoplastic Flask subjected to an uncertain heat, will crack the plaster investment, The metal when poured will probably not be retained, the teeth will be checked, and at the best a misfit is a certainty. By means of the Fabricator a casting can be made into the commonest glass bottle and fracture is impossible (*i. e.*, assuming a suitable alloy be used.) Without the aid of a thermometer temperature is ensured which will evaporate all moisture without danger of cracking model or investments, after the whole is elevated to a degree corresponding to the melting point of the metal employed; after cooling, the work of removal from the flask will necessitate but little labor, time, or skill, to make it fit for the mouth.

The whole process is clean, simple and elegant, a great portion of it intrusted to the least experienced pupil will result satisfactorily; in this respect it most favorably compares with every known system of Prosthesis. It is hoped that the Celluloid craze is totally consigned to a dishonored grave, but though a worthless thing, yet much valuable time and ability was spent in connection therewith, and many a good practice injured thereby; but such as

*Patent No. 304, 766; Sept. 9th, 1884.

used it did so in the belief that it obviated some of the inherent defects of rubber, a notable one being its non-conductivity. To these, this system is heartily commended. The Cheoplastic system is by no means recommended for universal use, as its value is only "*especial*" in the inferior maxillary—in that respect it has no equal; its weight (which, though, is imperceptible in the mouth of the patient) and a certain property of *vis inertia,* makes a denture remain in its place "*like a corpse in bed*;', a normal thermal condition is maintained, hence inflammation and excoriation is rarely experienced, a much less rapid absorption of the alveolar occurs, and a patient acquires such confidence that mastication is soon achieved. Much more might be said, but your space is limited, and the field has been well covered by Dr. Atkinson and Reese, to whom be all praise and honor.—*The Dental Advertiser.*

